# Usefulness of Selected Peripheral Blood Counts in Predicting Death in Patients with Severe and Critical COVID-19

**DOI:** 10.3390/jcm11041011

**Published:** 2022-02-15

**Authors:** Michał P. Pluta, Mateusz N. Zachura, Katarzyna Winiarska, Alicja Kalemba, Cezary Kapłan, Anna J. Szczepańska, Łukasz J. Krzych

**Affiliations:** 1 Department of Anaesthesiology and Intensive Care, Faculty of Medical Sciences in Katowice, Medical University of Silesia, Medykow 14 Street, 40752 Katowice, Poland; aszczepanska@sum.edu.pl (A.J.S.); lkrzych@sum.edu.pl (Ł.J.K.); 2 Emergency Medicine Department, St. Barbara’s Memorial Hospital No. 5 Trauma Center, Medyków 1 Square, 41200 Sosnowiec, Poland; 3 Students’ Scientific Society, Department of Anaesthesiology and Intensive Care, Faculty of Medical Sciences in Katowice, Medical University of Silesia, Medykow 14 Street, 40752 Katowice, Poland; mateusz.zachura@gmail.com (M.N.Z.); k.winiarska.97@gmail.com (K.W.); alicja.k.kalemba@gmail.com (A.K.); cezary.kaplan@gmail.com (C.K.)

**Keywords:** COVID-19, SARS-CoV-2, intensive care unit, complete blood count, neutrophil–lymphocyte ratio, prediction

## Abstract

Background. Immune dysregulation and hypoxemia are two important pathophysiological problems in patients with COVID-19 that affect peripheral blood count parameters. We hypothesized that assessment of the neutrophil–lymphocyte ratio (NLR) and red blood cell distribution width index (RDW-SD) could predict death in patients with severe and critical COVID-19. Methods. Seventy patients admitted to the intensive care unit (ICU) for COVID-19 acute respiratory failure were included in the study. RDW-SD and NLR on the day of ICU admission and peak values during the entire hospitalization were assessed. The primary endpoint was death before ICU discharge. Results. Patients who died had higher NLR on admission (20.3, IQR 15.3–30.2 vs. 11.0, IQR 6.8–16.9; *p* = 0.003) and higher RDW-SD (48.1 fL; IQR 43.1–50.5 vs. 43.9 fL; IQR 40.9–47.3, *p* = 0.01) than patients discharged from the ICU. NLR and RDW-SD values on ICU admission accurately predicted death in 76% (AUC = 0.76; 95%CI 0.65–0.86; *p* = 0.001; cut-off > 14.38) and 72% of cases (AUC = 0.72; 95%CI 0.60–0.82; *p* = 0.003; cut-off > 44.7 fL), respectively. Multivariable analysis confirmed that NLR > 14.38 on the day of ICU admission was associated with a 12-fold increased risk of death (logOR 12.43; 95%CI 1.61–96.29, *p* = 0.02), independent of other blood counts, clinical and demographic parameters. Conclusions. Neutrophil–lymphocyte ratio determined on the day of ICU admission may be a useful biomarker predicting death in patients with severe and critical COVID-19.

## 1. Introduction

In December 2019, the first cases of pneumonia caused by the new severe acute respiratory syndrome (SARS-CoV-2) coronavirus were diagnosed in China’s Hubei province [1]. During the coronavirus disease pandemic (COVID-19) declared by the World Health Organization, 364,191,494 people were infected and 5,631,457 (1.5%) had died by 30 January 2022 [2]. The pandemic has revealed significant financial disparities between countries, and in many of them has led to the collapse of public health systems [3]. In the face of the financial crisis and consequent impeded access to advanced diagnostic tools, it became necessary to review the utility of readily available and inexpensive laboratory parameters in prioritizing the admission of COVID-19 patients to a limited number of intensive care beds [4,5,6].

Patients with severe COVID-19 infection present two significant pathophysiologic problems: excessive inflammatory response (1) and hypoxemia (2). Each of these problems can potentially be reflected in the results of the baseline complete blood count (CBC). The inflammatory response stimulates neutrophil production and enhances lymphocyte apoptosis to a degree dependent on its severity. The neutrophil–lymphocyte ratio (NLR) may be a marker of immune dysregulation and systemic stress, providing potentially more valuable information than either parameter alone [7]. On the other hand, cellular hypoxia stimulates erythropoietin production, which accelerates the formation of red blood cells (RBCs) with increased volume (MCV), leading to an increase in red cell distribution width index (RDW). Additionally, excessive release of inflammatory cytokines inhibits the growth and shortens the survival time of RBCs, stimulating reticulocyte production, also increasing RDW [8,9].

It has been hypothesized that the assessment of these two biomarkers, NLR and RDW, may be a useful component of death risk stratification in severe and critical COVID-19 patients.

## 2. Materials and Methods

### 2.1. Study Design

This was a single-center observational study conducted in the medical ICU of a Polish University Hospital, which retrospectively evaluated the outcomes of CBC in patients admitted to the ICU between 10.2020 and 06.2021r with a diagnosis of severe and critical COVID-19 (i.e., second and third wave of COVID-19 in Poland). Due to the non-interventional, retrospective nature of the study, informed consent was not required from patients to participate [10]. To avoid potential influence on the results, the ICU treatment team had no knowledge of the planned data analysis.

This article was prepared in accordance with the STROBE (Strengthening The Reporting of Observational Studies in Epidemiology) reporting guidelines [11].

### 2.2. Patients

All patients hospitalized in the ICU for severe and critical COVID-19 (*n* = 73) were included in the study. COVID-19 infection was confirmed by a positive RT-PCR test. Criteria for severe COVID-19 were defined as: (1) SpO_2_ < 94% in air, (2) respiratory rate > 30/min, (3) ratio of arterial partial pressure of oxygen to inspired oxygen concentration (PaO_2_/FiO_2_) <300 mm Hg or (4) lung lesions involving >50% of the lung surface area [12]. Critical COVID-19 progressed to respiratory failure, septic shock, and/or multiple organ failure (MOF) [12]. ICU admission priority was determined by Society of Critical Care Medicine (SCCM) criteria adapted to local conditions, as recommended by the Polish Society of Anaesthesiology and Intensive Care (PTAiIT) [13]. 

Patients with a proliferative hematologic process (*n* = 1) and those requiring transfer to another center for extracorporeal blood oxygenation (*n* = 2) were excluded from the final analysis).

### 2.3. Clinical Data

Demographic and clinical data were collected, including age, gender, past medical history, priority of ICU admission according to SCCM recommendations, length of ICU hospitalization, time from admission to endotracheal intubation, degree of lung injury assessed by lung computed tomography (HRCT) analysis by an experienced radiologist, diagnosis of pulmonary embolism based on pulmonary artery angiography, methods of ventilation support (invasive and non-invasive), application of pharmacological treatment currently recommended for patients with COVID-19 by the Polish Agency for Health Technology Assessment and Tariffication, pharmacological circulatory support, extracorporeal therapies (continuous renal replacement therapy, cytokine absorbers, therapeutic plasma exchange) and ventilation in the prone position.

### 2.4. Laboratory Data

Blood for CBC was collected with a BD Vacutainer^TM^ system (Becton Dickinson, Franklin Lakes, NJ, USA) into EDTA tubes. The material was transferred to the laboratory according to local protocols for handling infectious material, where it was analyzed using a Sysmex XT-1800i automated hematology analyzer (Sysmex Corporation, Kobe City, Japan) immediately upon receipt. Standard hematological parameters were determined along with an automated leukocyte smear. The frequency of CBC was at the discretion of the attending physician and was not standardized. 

NLR calculation was not used in daily clinical practice. The hematology analyzer did not automatically calculate the NLR. The NLR calculation was performed retrospectively for the study. Then, based on the data from previous clinical studies and the proposal of Farkras J. [14], patients were classified into one of four groups of systemic stress severity considering only the NLR score at ICU admission: normal stress (NLR < 6), mild stress (NLR 6–9), moderate stress (NLR 9–18), severe stress (NLR > 18). In addition, the maximum NLR (maxNLR) and RDW-SD (maxRDW-SD) were determined from all CBC tests performed for the patient during hospitalization in the ICU.

### 2.5. Outcome

The endpoint was the patient’s death before ICU discharge.

### 2.6. Statistical Analysis

Statistical analysis was performed using procedures available in MedCalc Statistical Software version 18.2.1 (MedCalc Software bvba, Ostend, Belgium; http://www.medcalc.org, accessed on 1 October 2021). Quantitative variables were presented as the median and interquartile range (IQR). Qualitative variables were presented as absolute values and percentages. The difference between quantitative variables was assessed using analysis of variance or the Kruskal–Wallis test. For qualitative variables, the chi-square test or Fisher’s exact test were used when the group size was small (N ≤ 30). Statistical relationships for dichotomous variables were assessed by odds ratio (OR) analysis. Diagnostic accuracy was assessed by ROC curves and area under curve (AUC). Finally, a logistic regression model was created in which the dependent variable was death before ICU discharge, and the independent variables were those that differed between groups at *p* < 0.1 in simple analyses. Survival probability in terms of NLR on admission was subjected to Kaplan–Meier analysis and presented using log-rank tests and hazard ratio (HR) with 95% confidence intervals (95%CI).

The criterion for statistical significance was *p* < 0.05.

## 3. Results

The final analysis included 525 CBC results in 70 patients hospitalized in the ICU. The median age of the subjects was 66 years [IQR 60–71]. The median time between CBC determinations was 2 days [IQR 1–3]. Overall, 81% of patients had died by ICU discharge (*n* = 57). Detailed demographic and clinical data are shown in Table 1.

### 3.1. Peripheral Blood Leukocyte Parameters

Patients who died before ICU discharge had a statistically significantly lower LYMPH count (*p* = 0.003) and higher NEUT count (*p* = 0.005) on the day of ICU admission. The total WBC count was not significantly different between the groups (*p* = 0.4). The values of peripheral blood morphological parameters in the study group on the day of admission to the ICU are shown in Table 2.

The count of LYMPH on admission to the ICU predicted death in 74% (AUC = 0.74; 95%CI 0.62–0.84; *p* = 0.007) at the cut-off point ≤ 0.87 × 10^6^ L^−1^ with a sensitivity of 84% and specificity of 61%. The NEUT value at ICU admission showed no significant accuracy in predicting the risk of death (AUC = 0.63; *p* = 0.14).

### 3.2. Neutrophil–Lymphocyte Ratio (NLR)

**Figure 1:**
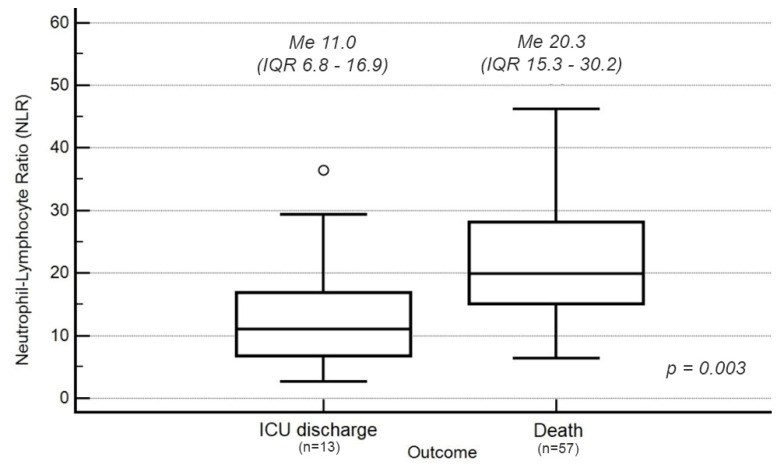
NLR values on admission to the ICU, and survival by the discharge from the ICU. The length of the rectangle represents the interquartile range (IQR), comprising the middle 50% of observations. The box is separated by a horizontal line that marks the median value (Me). It divides the quartile interval (Q) into two areas containing 25% of the observations. The whiskers connect the box with the largest and smallest values of the studied variable from the interval (Q1 − 1.5 × IQR; Q1) and (Q3; Q3 + 1.5 IQR), respectively. Dots indicate outliers.

The median NLR on ICU admission was 18.1 (IQR 12.0–27.7). Patients who died had a significantly higher NLR on admission compared to patients who survived (20.3, IQR 15.3–30.2 vs. 11.0, IQR 6.8–16.9; *p* = 0.003) (Figure 1).

**Figure 2:**
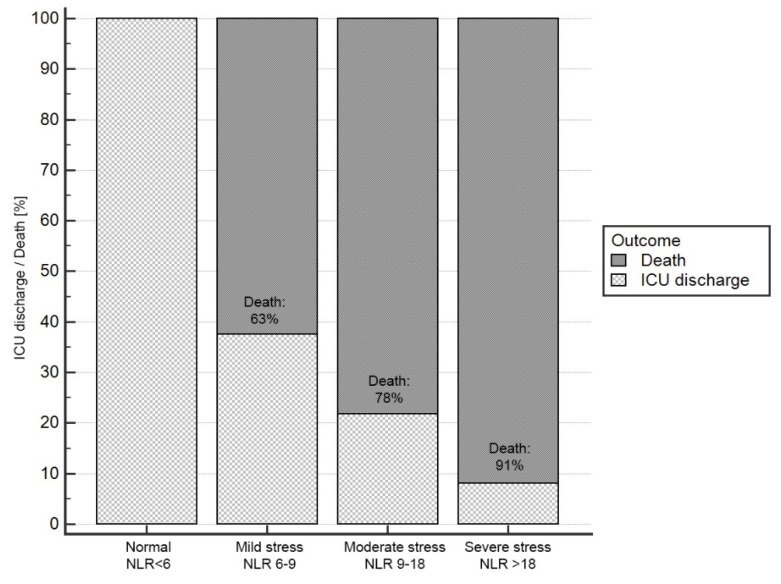
Final outcome of ICU treatment by systemic stress severity group (based on NLR index [14]).

After retrospective matching of patients to systemic stress severity group based on NLR, it was shown that mortality significantly increased with increased NLR. In the group of patients whose NLR on admission to the ICU was above 18 (severe stress group), 91% of patients died, which accounted for 60% of all deaths in the study population (Table 3) (Figure 2).

Patients in the higher systemic stress group (according to the NLR score) had a three-fold higher risk of death than patients classified in the lower group (OR = 3.32; 95%CI 1.50–7.36; *p* = 0.003).

NLR value on ICU admission predicted death with 76% accuracy (AUC = 0.76; 95%CI 0.65–0.86; *p* = 0.001) at NLR cut-off point > 14.38 with 77% sensitivity and 69% specificity. NLR value above the indicated cut-off point indicated a seven-fold increased risk of death (OR = 7.61; 95%CI 2.01–28.81; *p* = 0.002). 

When analyzing NLR values throughout the ICU stay, patients who ultimately died presented significantly higher maximum NLR (maxNLR) values during hospitalization (34.98, IQR 22.51–58.54 vs. 18.13, IQR 7.45–29.79; *p* = 0.001). A maxNLR value > 31.51 was associated with death in 79% of cases (AUC = 0.79; 95%CI 0.68–0.88; *p* < 0.001) with a sensitivity of 58% and specificity of 92%.

**Figure 3:**
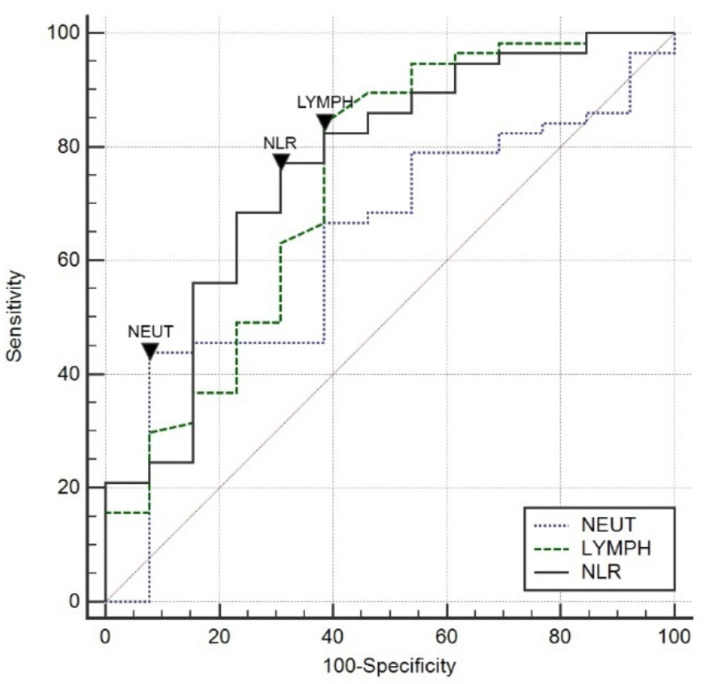
Comparison of ROC curves for selected leukocyte parameters measured on admission to the ICU.

A comparison of ROC curves for selected leukocyte parameters, determined on ICU admission, is shown in Figure 3.

### 3.3. Red Cell Distribution Width (RDW)

**Figure 4:**
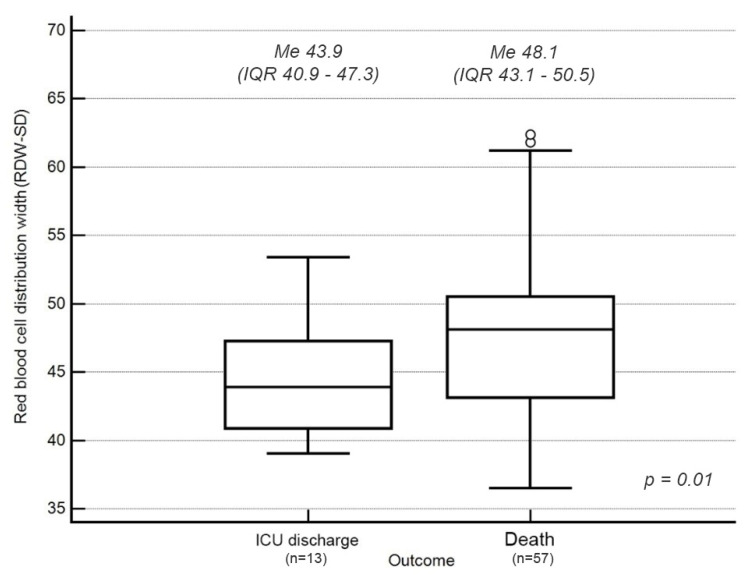
RDW-SD values at ICU admission and survival to ICU discharge. The length of the rectangle represents the interquartile range (IQR), comprising the middle 50% of observations. The box is separated by a horizontal line that marks the median value (Me). It divides the quartile interval (Q) into two areas containing 25% of the observations. The whiskers connect the box with the largest and smallest values of the studied variable from the interval (Q1 − 1.5 × IQR; Q1) and (Q3; Q3 + 1.5 IQR), respectively. Dots indicate outliers.

In terms of red blood cell parameters, patients who died had statistically significantly higher RDW-SD values on ICU admission compared to patients who survived prior to ICU discharge (48.1 fL; IQR 43.1–50.5 vs. 43.9 fL; IQR 40.9–47.3, *p* = 0.01) (normal laboratory range in the study center: 38.90–50 fL) (Figure 4)

An RDW-SD value on ICU admission above 44.7 fL accurately predicted death in 72% of cases (AUC = 0.72; 95%CI 0.60–0.82; *p* = 0.003) with a sensitivity of 70% and specificity of 69%. An RDW-SD score > 44.7 fL on ICU admission was associated with a greater than five-fold increased risk of death (OR = 5.29; 95%CI 1.43–19.56; *p* = 0.009). 

Similar to NLR, maximum RDW-SD (maxRDW-SD) values during ICU hospitalization were significantly higher in patients who had died by ICU discharge. A maxRDW-SD value > 46.8 fL predicted death in 76% of cases (AUC = 0.76; 95%CI 0.65–0.86; *p* = 0.006) with a sensitivity of 91% and specificity of 62%. 

### 3.4. Logistic Regression Model

A logistic regression model confirmed that NLR > 14.38 on ICU admission, regardless of other parameters (i.e., age, sex, degree of lung injury on HRCT, LYMPH, NEUT, and RDW-SD), was associated with a greater than 12-fold increased risk of death (logOR 12.43; 95%CI 1.61–96.29, *p* = 0.02 przy AUC = 0.90; 95%CI 0.79–0.97; *p* = 0.049).

### 3.5. Survival Analysis

**Figure 5:**
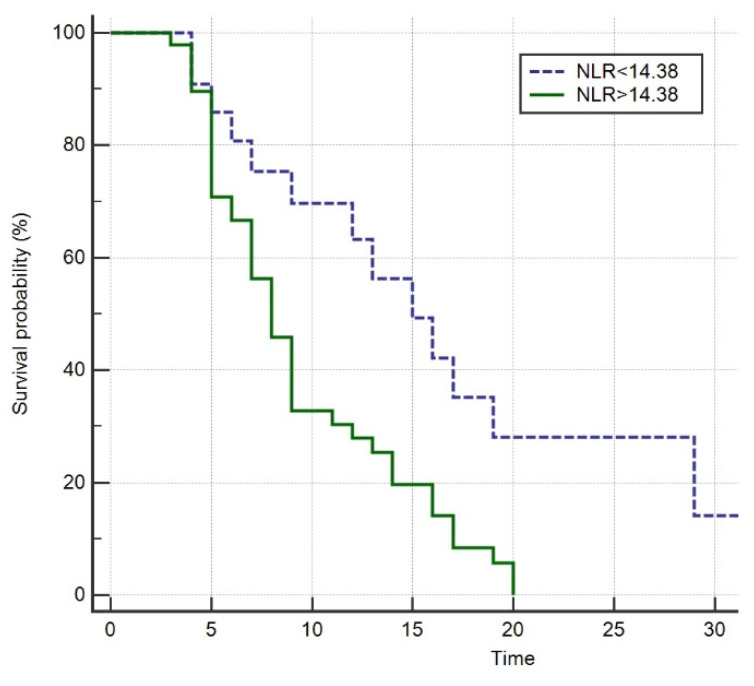
Survival probability in terms of NLR on admission.

Kaplan–Meier analysis showed that patients with an NLR > 14.38 on ICU admission had a significantly higher risk of death compared to patients with a lower NLR (log-rank test; *p* = 0.003; HR 2.29, 95%CI 1.35–3.88) (Figure 5).

## 4. Discussion

This single-center observational study demonstrated that the NLR value on ICU admission was a useful predictor of death in severe and critical COVID-19 patients, independent of RDW-SD and other clinical and demographic parameters. The study was retrospective, clinicians were not informed of the planned data analysis, and the NLR score was not routinely provided by the hospital laboratory. To our knowledge, no clinicians calculated the NLR for clinical decision making. 

Previous studies have shown a leukocyte pattern characteristic of COVID-19, i.e., higher NEUT and lower LYMPH [15]. Since inflammatory cytokines stimulate the maturation of T cells that destroy SARS-CoV-2-infected cells, lymphocytosis correlates with an increased risk of death. The percentage of CD8+ T cells is significantly lower in severe and critical COVID-19 patients, but unlike NEUT and LYMPH counts, lymphocyte subpopulation analysis is not currently routine clinical practice [16]. In our study, when considering the classification of systemic stress proposed by Farkras J. [14], patients in the NLR < 6 (normal stress) group survived before ICU discharge, although the degree of lung involvement on HRCT was not significantly different compared to the severe systemic stress group (NLR > 18), in which mortality was 91%. Multivariate analysis also identified NLR as a predictor of death independent of the degree of lung injury. These observations support the involvement of cytokine storms in the development of MOF in the course of COVID-19. However, it is worth noting that in our study, the degree of lung injury was subjectively assessed by the radiologist. When Liu F. et al. used artificial intelligence algorithms to evaluate HRCT images, they were more useful in risk stratification than any laboratory parameters, but such systems are not routinely used [17]. The NLR cut-off point for assessing the risk of disease progression or death varied between studies. Yang A. et al. showed that age < 49.5 years and NLR < 3.3 predicted hospital discharge after approximately 13.5 days of hospitalization (AUC 0.84; *p* < 0.05) [18]. Shang W. et al. identified NLR > 4.28 as predictive of severe COVID-19 (AUC = 0.74; *p* < 0.001) [19]. In the study by Yan X. et al. [20], NLR > 11.75 value predicted in-hospital death from any cause with very good accuracy (AUC = 0.94; *p* < 0.01). A recent meta-analysis by Ulloque-Badaracco JR et al., which included 61 studies (*n* = 15,522 patients), showed that an increase of one NLR unit was associated with a higher risk of disease progression (OR 6.22; 95%CI 4.93–7.84; *p* < 0.001) and a higher risk of in-hospital death from any cause (OR 12.6; 95%CI 6.88–23.06; *p* < 0.001) [21]. In our study, the optimal cut-off point on the ROC curve for predicting death corresponded to NLR > 14.38, and the AUC for NLR was higher than that for NEUT and LYMPH separately. Given the lack of cost associated with obtaining NLR values, the NLR parameter should be routinely provided to clinicians by the laboratory with CBC results. The usefulness of NLR has also been demonstrated in many other clinical situations [22,23]. 

In our study group, we noted an alarmingly high mortality rate (81%). However, by taking NLR as an indicator of disease severity, we included sicker patients in our study than other investigators (median NLR at admission 18.1; IQR 12.0–27.7). For example, in the cited meta-analysis by Ulloque-Badaracco JR et al., only 3 of 61 studies had a median NLR higher than 18.1, and the mortality reported in one of them was even higher than in our center (87.5%) [18]. The other authors did not disclose the number of deaths.

Our observations have important implications for daily clinical practice. NLR assessment can support the identification of patients at risk of COVID-19 progression to MOF and death, and therefore indicate the need for early eligibility for centers with extracorporeal support methods. On the other hand, in a situation of limited access to mechanical ventilation, the effective identification of patients who will not benefit from the implementation of methods and techniques reserved for the ICU will allow the targeting of human and equipment resources to patients potentially promising for recovery. This type of triage is a key component of incident management where the number of casualties exceeds available forces and resources, familiar from disaster and battlefield medicine [24].

### Limitations

Our study has several limitations. First, it was a single-center observation, so the results cannot be uncritically extrapolated to other populations. Second, the NLR value may be affected by the use of exogenous glucocorticosteroids [14,25]. Although the management of dexamethasone use in our ICU was consistent with the AOTM recommendations, we cannot guarantee that the recommendations were followed prior to ICU admission [26]. Third, we did not analyze other potential causes of lymphopenia and lymphocytosis or neutropenia and neutrocytosis, but a priori excluded patients suspected of having a proliferative hematologic process from our analysis. Fourth, although many publications have referred to the usefulness of NLR in COVID-19, few of the studies involved such an extremely severe group of patients. 

## 5. Conclusions

The neutrophil–lymphocyte ratio determined on the day of intensive care unit admission may be a useful biomarker for predicting death in patients with severe and critical COVID-19, even after accounting for other relevant medical variables. 

## Figures and Tables

**Table 1:** Selected demographic and clinical data.

Parameter	Survival ^1^	Death	*p*
	(*n* = 13)	(*n* = 57)	
Age (years)			
Median (IQR)	57 (53–67)	67 (61–72)	0.01
Sex			
male, *n* (%)	4 (31%)	43 (75%)	0.002
female, *n* (%)	9 (69%)	14 (25%)	0.002
Past medical history			
Obesity, *n* (%)	8 (61%)	37 (65%)	0.8
Hypertension, *n* (%)	8 (61%)	32 (56%)	0.7
Diabetes, *n* (%)	2 (15%)	11 (19%)	0.7
Chronic kidney disease, *n* (%)	2 (15%)	6 (11%)	0.6
COPD, *n* (%)	1 (8%)	5 (9%)	0.9
CAD, *n* (%)	4 (31%)	14 (25%)	0.7
Hypothyroidism, *n* (%)	1 (8%)	3 (5%)	0.7
Stroke, *n* (%)	-	2 (4%)	-
Duration of ICU hospitalization (days)			
Median (IQR)	11 (8–15)	8 (5–13)	0.3
% lung injury ^2^			
Median (IQR)	70 (26–79)	80 (70–90)	0.03
Pulmonary embolism, *n* (%)	1 (8%)	6 (10%)	0.7
ICU admission priority ^3^			
1, *n* (%)	11 (85%)	47 (82%)	<0.001
2, *n* (%)	2 (15%)	10 (18%)	0.02
Ventilation			
HFNOT, *n* (%)	8 (62%)	31 (54%)	<0.001
NIV, *n* (%)	11 (85%)	35 (61%)	<0.001
IMV, *n* (%)	8 (62%)	56 (98%)	<0.001
Selected arterial blood gas parameters ^4^			
pH, Median (IQR)	7.37 (7.35–7.44)	7.35 (7.27–7.42)	0.1
pO_2_, Median (IQR)	75 (63–84)	67 (51–88)	0.5
pCO_2_, Median (IQR)	40 (32–50)	39 (32–50)	0.9
% SaO_2_, Median (IQR)	94 (90–96)	91 (86–96)	0.2
Time from hospital admission to intubation (days)			
Median (IQR)	2.5 (1.5–4)	4.5 (3–10)	0.1
Prone position, *n* (%)	12 (92%)	45 (79%)	0.3
Pharmacotherapy ^5^			
NMBA, *n* (%)	7 (54%)	48 (84%)	0.02
Dexamethasone, *n* (%)	13 (100%)	57 (100%)	<0.001
Remdesivir, *n* (%)	4 (31%)	13 (23%)	0.1
Tocilizumab, *n* (%)	4 (31%)	15 (26%)	0.7
Pharmacol. support of the cardiovasc. system			
Adrenaline, *n* (%)	1 (8%)	46 (81%)	<0.001
Norepinephrine, *n* (%)	8 (62%)	57 (100%)	<0.001
Argipressin, *n* (%)	1 (8%)	26 (46%)	0.01
Dopamine, *n* (%)	-	5 (9%)	-
Dobutamine, *n* (%)	1 (8%)	11 (19%)	0.3
Milrinone, *n* (%)	-	2 (4%)	-
Extracorporeal Therapies			
TPE, *n* (%)	2 (15%)	5 (9%)	0.5
CRRT, *n* (%)	3 (23%)	25 (44%)	0.2
Cytokine adsorbers, *n* (%)	1 (8%)	7 (12%)	0.6

^1^ By the discharge from the Intensive Care Unit; ^2^ based on CT; ^3^ according to the guidelines of the Society of Critical Care Medicine and the Polish Society of Anaesthesiology and Intensive Care; ^4^ upon admission to ICU; ^5^ including prior to admission to the Intensive Care Unit; IQR, interquartile range; COPD, chronic obstructive pulmonary disease; CAD, coronary artery disease; HFNOT, high-flow nasal oxygen therapy; NIV, non-invasive ventilation; IMV, invasive mechanical ventilation; NMBA, neuromuscular blocking agents; TPE, therapeutic plasma exchange; CRRT, continuous renal replacement therapy.

**Table 2:** Morphological parameters of peripheral blood determined on the day of admission to the Intensive Care Unit.

Parameter	All (*n* = 70) Me (IQR)	Survival ^1^ (*n* = 13) Me (IQR)	Death (*n* = 57) Me (IQR)	*p*
WBC (×10^9^ L^−1^)	13.0 (9.3–16.7)	11.3 (9.1–15.7)	13.1 (9.3–19.1)	0.4
RBC (×10^12^ L^−1^)	4.1 (3.6–4.7)	4.1 (3.7–5.0)	4.1 (3.5–4.7)	0.3
HGB (g dL^−1^)	12.7 (10.9–14.1)	13.3 (10.5–15.3)	12.6 (11.1–14.0)	0.3
Hematocrit (%)	37 (34–43)	40 (31–45)	37 (35–43)	0.9
MCV (fL)	91.1 (87–98)	89 (85–91)	92 (88–98)	0.02
MCH (pg)	30 (29–32)	30 (29–31)	31 (30–33)	0.05
MCHC (g dL^−1^)	34 (33–34)	34 (33–34)	34 (33–34)	0.8
PLT (×10^6^ L^−1^)	226 (176–305)	254 (226–370)	210 (168–298)	0.07
LYMPH (%)	4.8 (3.2–6.8)	7.3 (5.4–11.7)	4.5 (3.0–5.9)	0.003
LYMPH (×10^6^ L^−1^)	0.60 (0.42–0.87)	1.0 (0.5–1.4)	0.5 (0.4–0.8)	0.007
MONO (%)	3.45 (2.5–4.9)	4.7 (3.1–5.2)	3.3 (2.4–4.6)	0.1
MONO (×10^6^ L^−1^)	0.43 (0.27–0.63)	0.5 (0.4–0.7)	0.4 (0.2–0.6)	0.2
NEUT (%)	89.2 (85.1–91.7)	81.8 (80.2–89.6)	89.8 (87.4–92.2)	0.005
NEUT (×10^6^ L^−1^)	11.5 (7.9–15.2)	9.0 (7.5–12.8)	11.7 (8.5–17.9)	0.1
EOS (%)	0.0 (0.0–0.1)	0.0 (0.0–0.2)	0.0 (0.0–0.0)	0.3
EOS (×10^6^ L^−1^)	0.0 (0.0–0.01)	0.0 (0.0–0.0)	0.0 (0.0–0.1)	0.4
BASO (%)	0.2 (0.1–0.2)	0.2 (0.1–0.3)	0.1 (0.1–0.2)	0.2
BASO (×10^6^ L^−1^)	0.02 (0.01–0.04)	0.02 (0.01–0.04)	0.02 (0.01–0.03)	0.7
RDW-SD (fL)	46.9 (42.9–49.8)	43.9 (40.9–47.3)	48.1 (43.1–50.5)	0.01
PCT (%)	0.24 (0.20–0.33)	0.27 (0.24–0.41)	0.23 (0.20–0.33)	0.1
MPV (fL)	10.8 (10.2–11.7)	10.5 (9.7–11.4)	10.8 (10.2–11.7)	0.3
PDW (%)	12.8 (11.1–14.4)	12.1 (10.8–13.6)	12.9 (11.3–15)	0.4

^1^ By the discharge from the Intensive Care Unit; Me, median; IQR, interquartile range; WBC, white blood cells; RBC, red blood cells; HGB, hemoglobin; MCV, mean corpuscular volume; MCH, Mean corpuscular hemoglobin; MCHC, mean corpuscular hemoglobin concentration; PLT, platelet; LYMPH, lymphocytes; MONO, monocytes; NEUT, neutrophils; EOS, eosinophils; BASO, basophils; RDW-SD; red blood cell distribution width—standard deviations; PCT, plateletcrit; MPV, mean platelet volume; PDW, platelet distribution width.

**Table 3:** Values of selected clinical and demographic parameters in systemic stress severity groups (based on [14]).

Variable	Group				*p*
	Normal	Mild	Moderate	Sever	
	Stress	Stress	Stress	Stress	
	NLR < 6	NLR 6–9	NLR 9–18	NLR > 8	
*n*, (%)	2 (3%)	8 (11%)	23 (33%)	37 (53%)	<0.001
Age (years)					
Median (IQR)	54 (54–78)	62 (56–69)	67 (60–71)	65 (61–73)	0.7
Sex					
male, *n* (%)	-	5 (7%)	16 (23%)	25 (36%)	<0.001
female, *n* (%)	2 (3%)	3 (4%)	7 (10%)	11 (16%)	0.03
Duration of hospitalization in ITU (days)					
Median (IQR)	15 (4–26)	12 (8–14)	12 (6–17)	8 (5–9)	0.2
% lung injury ^1^					
Median (IQR)	75 (60–90)	75 (65–85)	85 (70–90)	80 (70–80)	0.4
Pulmonary embolism, *n* (%)	-	1 (1%)	3 (4%)	3 (4%)	0.8
ICU admission priority ^2^					
1, *n* (%)	2 (3%)	5 (7%)	21 (30%)	30 (43%)	<0.001
2, *n* (%)	-	3 (4%)	2 (3%)	7 (10%)	0.2
Ventilation					
HFNOT, *n* (%)	2 (3%)	5 (7%)	13 (19%)	19 (27%)	0.6
NIV, *n* (%)	2 (3%)	5 (7%)	15 (21%)	24 (34%)	0.8
IMV, *n* (%)	1 (1%)	6 (9%)	21 (30%)	36 (51%)	0.03
Prone position, *n* (%)	2 (3%)	8 (11%)	18 (26%)	29 (41%)	0.4
Pharmacol. support of the cardiovasc. system					
Adrenaline, *n* (%)	-	5 (7%)	15 (21%)	27 (39%)	0.2
Norepinephrine, *n* (%)	1 (1%)	6 (9%)	21 (30%)	37 (53%)	0.006
Argipressin, *n* (%)	-	2 (3%)	11 (16%)	14 (20%)	0.4
Dopamine, *n* (%)	-	-	2 (3%)	3 (4%)	0.8
Dobutamine, *n* (%)	-	2 (3%)	2 (3%)	8 (11%)	0.5
Milrinone, *n* (%)	-	-	-	2 (3%)	0.6
Extracorporeal therapies					
TPE, *n* (%)	-	1 (1%)	3 (4%)	3 (4%)	0.9
CRRT, *n* (%)	-	4	7 (10%)	17 (24%)	0.4
Cytokine adsorbers, *n* (%)	-	3 (4%)	1 (1%)	4 (6%)	0.08
Death before discharge from ICU	-	5 (7%)	18 (26%)	34 (49%)	0.003

^1^ Based on CT; ^2^ according to the guidelines of the Society of Critical Care Medicine and the Polish Society of Anaesthesiology and Intensive Care; IQR, interquartile range; HFNOT, high-flow nasal oxygen therapy; NIV, non-invasive ventilation; IMV, invasive mechanical ventilation; TPE, therapeutic plasma exchange; CRRT, continuous renal replacement therapy.

## Data Availability

Data are available from the authors of the study.
